# Adult Niemann-Pick disease type C in France: clinical phenotypes and long-term miglustat treatment effect

**DOI:** 10.1186/s13023-018-0913-4

**Published:** 2018-10-01

**Authors:** Yann Nadjar, Ana Lucia Hütter-Moncada, Philippe Latour, Xavier Ayrignac, Elsa Kaphan, Christine Tranchant, Pascal Cintas, Adrian Degardin, Cyril Goizet, Chloe Laurencin, Lionel Martzolff, Caroline Tilikete, Mathieu Anheim, Bertrand Audoin, Vincent Deramecourt, Thierry Dubard De Gaillarbois, Emmanuel Roze, Foudil Lamari, Marie T. Vanier, Bénédicte Héron

**Affiliations:** 1 Department of Neurology, Reference Center for Lysosomal Diseases (CRLM), UF Neuro-Genetics and Metabolism, Hôpital Pitié-Salpêtrière, 47–87, Boulevard de l’Hôpital, 75013 Paris, France; 2 Department of Pediatrics, Helios Clinic Sangerhausen, Sangerhausen, Germany; 3 Neurologic/Cardiologic Diseases Unit, Lyon East Biochemistry/Molecular Biology Department, CBPE,Hospices Civils de Lyon, Lyon, France; 4 Department of Neurology, Montpellier CHU, Gui De Chauliac Hospital, Montpellier, France; 5 Clinical Neurosciences, Timone CHU, Marseille Hospital, Marseille, France; 6 Department of Neurology, Hautepierre Hospital, Strasbourg, France; 7 Reference Centre for Neuromuscular Pathologies, Toulouse CHU, Pierre Paul Riquet Hospital, Toulouse, France; 8 Department of Neurology and Movement Disorders, Roger Salengro Hospital, Lille, France; 9 Centre de Référence Neurogénétique, Service de Génétique, Hôpital Pellegrin, University Hospital of Bordeaux and Laboratoire MRGM, INSERM U1211, University of Bordeaux, Bordeaux, France; 10 Department of Neurology, Pierre Wertheimer Neurology Hospital, Lyon, France; 11 Department of Internal Medicine, Hôpital Emile Muller, Mulhouse and South Alsace Regional Hospital Group, Mulhouse, France; 12 Hospices Civils de Lyon, Neuro-Ophthalmology and Neurocognition, Hôpital Neurologique Pierre Wertheimer, Lyon I University, and CRNL INSERM U1028 CNRS UMR5292, ImpAct Team, F-69676 Bron, France; 13 CRMBM UMR 7339, CNRS, Aix-Marseille Université, Marseille, France; 14 University of Lille, INSERM, CHU Lille, Degenerative & Vascular Cognitive Disorders, Lille, France; 15 St André Clinic, Reims, France; 16 Sorbonne UPMC University, INSERM U 1127, and the Institute for the Brain and Spinal Cord, Paris, France; 17 Department Metabolic Biochemistry and GRC 13-Neurometabolism-UPMC, Hôpital Pitié-Salpêtrière, Paris, France; 18 INSERM U820, Lyon, France; 19 Laboratoire Gillet-Mérieux, CBPE, Hospices Civils de Lyon, Lyon, France; 20 Reference Centre for Lysosomal Diseases (CRML), Department of Pediatric Neurology, and Sorbonne Université, GRC n°19, Pathologies Congénitales du Cervelet-LeucoDystrophies, AP-HP, Hôpital Armand Trousseau, F-75012 Paris, France; 21 Institute of Genetics and Molecular and Cellular Biology (IGBMC), INSERM-U964, Strasbourg University, Illkirch, France; 22 Strasbourg Federation of Translational Medicine (FMTS), Strasbourg University, Strasbourg, France; 23 APHM, Hôpital de la Timone, Clinical Neurosciences, Department of Neurology, Marseille, France

**Keywords:** Niemann-pick disease type C, Adult-onset, Epidemiology, Miglustat, Efficacy, Safety, France

## Abstract

**Background:**

Niemann-Pick disease type C (NP-C) is a neurodegenerative lysosomal lipid storage disease caused by autosomal recessive mutations in the *NPC1* or *NPC2* genes. The clinical presentation and evolution of NP-C and the effect of miglustat treatment are described in the largest cohort of patients with adolescent/adult-onset NP-C studied to date.

**Methods:**

Observational study based on clinical chart data from adult patients with NP-C (> 18 year old) diagnosed in France between 1990 and 2015. Retrospective data from patients at diagnosis, onset of miglustat therapy (if applicable), and last follow up were analysed.

**Results:**

In France, patients with an adolescent-adult neurological form constituted approximately 25% of all NP-C cases diagnosed during the study period. Forty-seven patients (46 with NP-C1 and one with NP-C2; 53% female) were included. Mean ± SD (range) ages at neurological onset and diagnosis were 23.9 ± 12.5 (8–56) years and 34 ± 13.5 (15–65) years, respectively. At presentation, patients mainly had 1) impaired gait due to cerebellar ataxia and/or dystonia, 2) and/or cognitive/behavioural manifestations, 3) and/or psychotic signs. Initially, almost half of patients had only one of the above three neuro-psychiatric manifestations. Vertical supranuclear gaze palsy, usually occurring without patient complaint, was only detected on careful clinical examination and was recorded in most patients (93%) at the time of diagnosis, several years after neurological onset. Thirty-seven patients (79%) received miglustat, among whom seventeen (46%) continued beyond 2 years (at last follow up) to a maximum of 9.8 years. Eight patients (22%) discontinued treatment early due to side effects (*n* = 3) or perceived lack of efficacy (*n* = 5).Miglustat treatment duration correlated significantly with reduced neurological worsening (*p* < 0.001). Treatment for≥2 years was associated with improved patient survival (*p* = 0.029). Good responses to miglustat were associated with less severe neurological disability at the start of miglustat treatment (*p* = 0.02).

**Conclusion:**

The proportion of adolescent/adult-onset NP-C cases diagnosed in France increased 2.5-fold since 2009 compared with the 2000–2008 period due to improved awareness. Adolescent/adult-onset NP-C frequently presented initially with a non-specific isolated neuro-psychiatric manifestation (motor, cognitive or psychotic). Patients with less severe neurological disability responded better to miglustat therapy.

**Electronic supplementary material:**

The online version of this article (10.1186/s13023-018-0913-4) contains supplementary material, which is available to authorized users.

## Background

Niemann Pick disease type C (NP-C) is a neurovisceral lysosomal storage disorder caused by autosomal recessive mutations in the *NPC1* (≥95% of cases) or the *NPC2* gene and is characterized by impaired trafficking of cholesterol and sphingolipids (reviewed in [1, 2]). The incidence of NP-C has been estimated at 1/100 000 to 1/120 000 live births based on diagnosed cases, but is likely higher [3]. The first symptoms are often visceral (especially in children), but in close to 90% of cases NP-C is primarily associated with progressive and severe neurological deterioration.

The age at presentation of NP-C is highly variable, and the clinical spectrum of the disease ranges from a perinatal, rapidly progressive systemic fatal disorder featuring acute liver or respiratory failure to an adult-onset chronic neurodegenerative form [4–11]. Aside from the perinatal systemic fatal form, the age at neurological onset and type of initial neurological manifestations are largely predictive of disease severity and indicative of life expectancy [2, 6, 12, 13]. These observations led to an early proposal in the 1990s [14] to classify NP-C into four main forms based on the age at onset of first neurological symptoms: early infantile- (onset at < 2 years of age), late infantile- (2–6 years), juvenile- (6–15 years), and adult- (≥15 years) onset NP-C. A small subset of patients suffering from isolated systemic disease (e.g. prolonged neonatal cholestatic jaundice, (hepato)splenomegaly) constitutes an intermediate ‘in waiting’ category, until the patient enters one of the above neurological forms. Of note, thus far, only a handful of such patients with proven NP-C have remained free of neurological manifestations even in late life [15], but these cases could be overlooked [16]. The classification of NP-C into these four neurological forms has proven more useful in clinical practice than the one based on age of first symptom, and has been followed in recent large natural history studies [6, 10, 11]. The NP-C clinical spectrum, however, is a continuum and there are overlaps between the neurological forms, particularly between the late-infantile and (early) juvenile forms, and the (late) juvenile/(early) adult forms. Increased knowledge on the natural history of NP-C, especially in relation to early signs and symptoms, may warrant a reappraisal of minor features of the historic classification. Indeed, since 2012, there has been an increasing trend to speak of an adolescent/adult neurological onset form (although keeping the same age at onset).

For a long time NP-C was primarily considered a paediatric disease, although cases with an adult onset had been described in the 1980s [17, 18]. Larger adult-onset patient cohorts have since been well documented [19–21] and adult-onset NP-C patients have increasingly been detected and diagnosed in recent years. They present with a different and variable clinical phenotype that frequently features a range of motor disorders (e.g., ataxia), cognitive decline, psychiatric symptoms (e.g. schizophrenia-like psychosis), and vertical supranuclear gaze palsy (VSGP), often without a systemic component [22]. Whilst a clinical NP-C suspicion index (SI) has been developed and proven effective in identifying patients with a high risk of the disease [23], the heterogeneity of neurological manifestations combined with the complexity of specific laboratory tests has made it difficult for clinicians to know when to test for NP-C in adult patients. The filipin test requires a skin fibroblast culture and an experienced laboratory to provide reliable findings, and complementary sequencing of the *NPC1* and *NPC2* genes is often necessary to confirm a diagnosis in adult patients [5, 24]. Gene testing alone may fall short due to difficult interpretation of observed genetic variants [25]. Together, these factors have led to long delays to diagnosis. The recent emergence of sensitive plasma biomarkers (such as cholestane-3β,5α,6β-triol and the coupled study of lysosphingomyelin-509 with lysosphingomyelin) has allowed more systematic disease screening and, in conjunction with the technical progress of genetic testing (still mandatory for confirmation), has led to a paradigm shift in the diagnosis of NP-C [3, 25, 26]. However, a lack of awareness of NP-C continues to contribute to the long-standing under-diagnosis of the disease among adults in general neurology and psychiatry.

Miglustat was approved for neurological manifestations of NP-C in the EU in 2009, and currently remains the only approved targeted therapy for the disease. This iminosugar-based agent is a competitive inhibitor of glucosylceramide synthase and is thought to prevent ganglioside accumulation in the brain, although its mode of action is likely more complex [27]. In clinical trials and early studies, miglustat has been shown to slow or stabilize progressive neurological manifestations in children and adults with NP-C [28–31]. While the efficacy of this agent has been further documented in more recent case series and cohort studies [32–38], there are few published analyses of its long-term impact on neurological progression in adult NP-C.

We report findings from a retrospective study of all adult NP-C patients diagnosed and followed up in French hospitals between 1990 and end of 2015. This cohort provides insight into the epidemiology of NP-C in France, particularly regarding the adolescent/adult form, and constitutes the largest series of patients with late-onset NP-C reported to date. We focused on the semiology and evolution of early and late neurological features, and evaluated the long-term effects of miglustat on neurological disabilities and survival by comparing miglustat-treated patients with non miglustat-treated patients.

## Methods

### Patients and study design

This was an observational, retrospective study of all adult NP-C patients aged > 18 years as at the end of 2015 who had neurological symptoms and whose diagnosis had been reported to the French Reference Center for Lysosomal Diseases (CRML). All included patients were aged ≥15 years when a diagnosis of NP-C was confirmed, except for patient 6, who was diagnosed at 3 months of age following severe splenomegaly with transient neonatal icterus, and who showed his first neurological manifestations at the age of 12 years. Diagnoses were based on filipin staining (with until 2009, a combined study of the rate of LDL-induced cholesteryl ester formation) [14, 24]) and/or*NPC1* and *NPC2* genetic analysis by MTV or PL at the Gillet-Mérieux Laboratory in Lyon-South or Lyon-East University Hospitals, France.

### Clinical questionnaires and neurological disability assessment

Clinical questionnaires were sent to all clinicians who diagnosed and/or followed up adult NP-C patients. The questionnaire focused on the semiology and time-course of neurological and psychiatric manifestations, and on the severity and progression of neurological disability, but also requested information regarding miglustat treatment (timing/duration and dose). In assessing age at neurological onset, manifestations including VSGP, hearing loss, and cognitive developmental deficits were excluded because: 1) VSGP is a clinical sign that occurs without patient complaint and its onset cannot accurately be determined; 2) hearing loss often occurs very early in the course of the disease, sometimes decades before other neurological symptoms, and was not considered a good marker of neurodegeneration onset; and 3) the association between cognitive developmental symptoms and initial neurodegeneration was not considered significant since most patients with intellectual disability exhibited other neurological signs only in adulthood and show a far better prognosis than classical infantile or juvenile forms of NP-C.

Neurological disability was assessed based on retrospective clinical chart information recorded at diagnosis, at commencement of miglustat therapy, and at miglustat discontinuation or last follow up, using a dedicated clinical disability scale [8] in its modified form [39]. This measure evaluates patient ambulation (max. 5 points), manipulation (max. 4 points), language (max. 5 points), swallowing (max. 4 points), ocular motor movement (max. 3 points) and epilepsy (max. 3 points), with a maximal total score of 24 points. The zero point in all domains indicates no symptoms.

Letters from the CRML (Paris, France) were sent to inform each patient about collection of data from their clinical charts, and included relevant contact details for provision of further information or for patients to refuse participation. Local ethics committee approval of the study was obtained from the CPP – Ile-de-France.

### Data analysis

Data analyses were mainly descriptive in nature based on observed data for all variables, with no imputation for missing data values. Kaplan-Meier time-to-event analyses were performed with SPSS® software version 21 for each key neurological manifestation. The time to event was defined as the period between overall ‘neurological onset’ and onset of the specific neurological manifestation of interest.

For other tests, data analyses were conducted using SAS® software version 9.3. Linear regression models were used to evaluate factors associated with change from baseline in total disability score and scores for each functional domain. Regression models were constructed using change in disability score as the dependent variable and delay from diagnosis to last follow up, duration of miglustat treatment, and clinical score at diagnosis as explicative variables. Times to severe disability scale score events in each of four functional domains (ambulation, manipulation, language and swallowing) were calculated using non-parametric, censored Kaplan-Meier time-to-event analyses. Median and 95% confidence interval (CI) time between onset of first dysfunction (per domain) and severe score events were determined. For patients who did not have severe score events, ‘time until last contact’ was used as censored observations. Time from diagnosis to death was also assessed: the Kaplan Meier curves for this analysis were truncated when approximately 10% of patients were still under observation in each group, due to low relevance of graphical representation based on limited patient numbers beyond this time point. The log-rank test was used to compare times to events between treatment groups. The Mann–Whitney test was used to compare patients categorized as good responders and poor responders to miglustat. An alpha-error cut-off point of 0.05 was considered in all statistical testing.

## Results

### General patient and disease characteristics: Late-onset cases in the French NP-C cohort

A total of 173 patients with NP-C referred from French hospitals (who might have variable ethnic/geographic origin) were diagnosed with NP-C during a 26-year observation period (1990–2015). Overall, the present study included 45 neurologically symptomatic adult patients from the French NP-C cohort and two further patients also seen in the Paris CRML but who were initially diagnosed in Switzerland [40].

In the French cohort, 35 (20%) patients (only eight of whom were diagnosed before 2009) had neurological onset between 15 and 56 years of age and clearly had the adult form of NP-C. Eight further patients (5%), who were 21–38 years old at last follow up, had developed minor initial symptoms aged 12–14 years with frank symptoms only appearing later, and exhibited a slow rate of neurological deterioration similar to that of classic adult-onset NP-C. These patients were considered to represent an early-adolescent neurological form rather than the known ‘classical’ juvenile form. An additional two (1%) slowly progressing patients who were 21 and 31 years old at last follow-up were also included. One (patient 1) had shown isolated slowly progressive ataxia for 7 years before VSGP was recognized, allowing diagnosis. The other (patient 2) attended school without any problem but showed mild clumsiness from the age of 10 years followed by overt neurological problems at the age of 17 years*.* Long-term follow up of the 10 patients with neurological onset before 15 years of age indicated that none had died before 29 years, which is in contrast to classical juvenile-onset patients, the majority of whom have been reported to die before 25 years of age [2].

Overall, we consider that all adult patients included in this study represent a wider adolescent/adult onset form of NP-C compared with the classical definition. For this reason we did not perform subgroup analyses based on age at neurological onset. Of note, one additional adult patient (currently 24 years old) who was diagnosed at the age of 3 years due to splenomegaly still shows no neurological symptoms, and was therefore not included in the study.

### Demographics and disease milestones

**Fig. 1:**
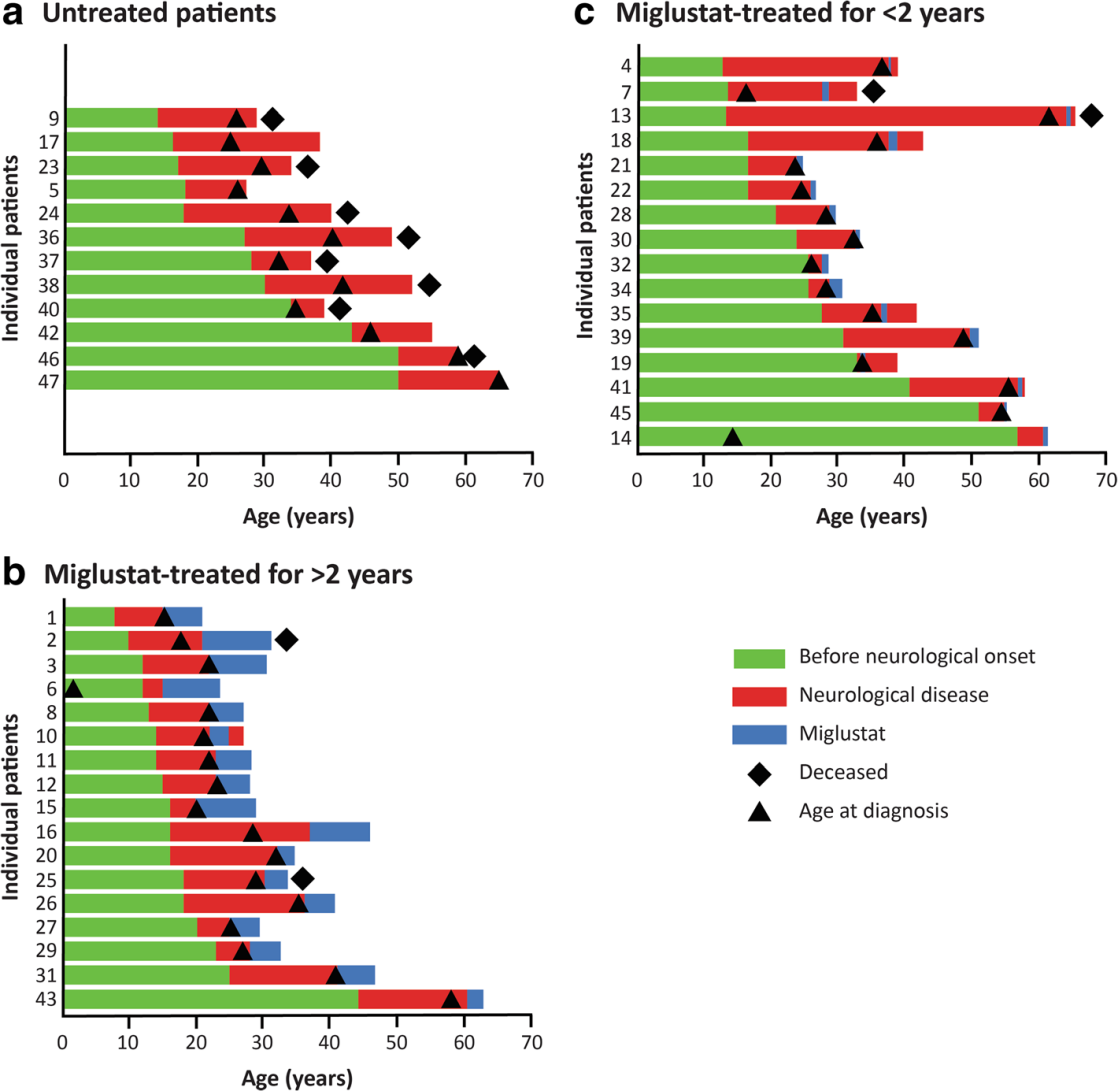
Schematic overview of the NP-C cohort with adolescent/adult neurological onset. Patients were divided in three categories : untreated (**a**), miglustat-treated for < 2years (**b**), miglustat-treated for > 2 years (**c**). See Additional file 1: Table S1 for details and further information

Figure 1 illustrates individual patient lifespans, periods before and during neurological manifestations, age at diagnosis, and where applicable, period of miglustat treatment for all included patients grouped according to their miglustat treatment status. Patients’ main individual details (including mutations and references to earlier reports in which some of the patients have been included) are provided in Additional file 1: Table S1**.** The number and proportion of male and female patients was approximately equal. One-quarter had affected siblings, and consanguineous family histories were recorded in 13% of patients (Table 1). Filipin testing had been performed in the Lyon laboratory for 41 unrelated patients, 28 (68%) of whom displayed a typical profile (13 ‘classic’, 15 ‘intermediate’) and 13 (32%) of whom had a variant profile [24]. Mutations in the *NPC1* gene were identified in 46 patients, and in the *NPC2* gene for one patient.

The mean (median) age at neurological onset was 23.9 (18.0) years, and the mean (median) age at diagnosis was 34.0 (31.0) years (Table 1). Twelve patients died during the 26-year study observation period, most commonly due to aspiration pneumonia resulting from severe dysphagia: 9/37 (24%) patients with clear adult-onset disease, and 3/10 (30%) patients with early-adolescent neurological onset. The mean (median) overall age at death was 41.5 (38.0) years (range 29.0–64.0 years): 33–64 years for those with clear adult onset and 29–32 years for those with early-adolescent onset. The mean (median) age at last follow up or death was 38.5 (35.0) years.

### Clinical phenotype: Disease manifestations and time course

Three patients exhibited intellectual disability (ID) and eight showed mild learning disabilities (LD) before onset of clinical neurological deterioration. Severe/marked hepatosplenomegaly (HSMG) was explored during the first years of life in seven patients, and resulted in early diagnosis of NP-C in one patient. Ages at neurological onset of NP-C were similar in patients with ID/LD (mean 21.4 years) compared with those without ID/LD (mean 24.8 years; *p* = 0.44), and in patients with childhood HSMG (mean, 19.1 years) compared with those without childhood HSMG (mean, 24.7 years; *p* = 0.28). Diagnostic work up conducted *after* neurological onset revealed only mild hepatomegaly (in 17/37 [46%] patients) and/or splenomegaly (in 27/40 [68%] patients), which was mainly detected by abdominal echography.

**Fig. 2:**
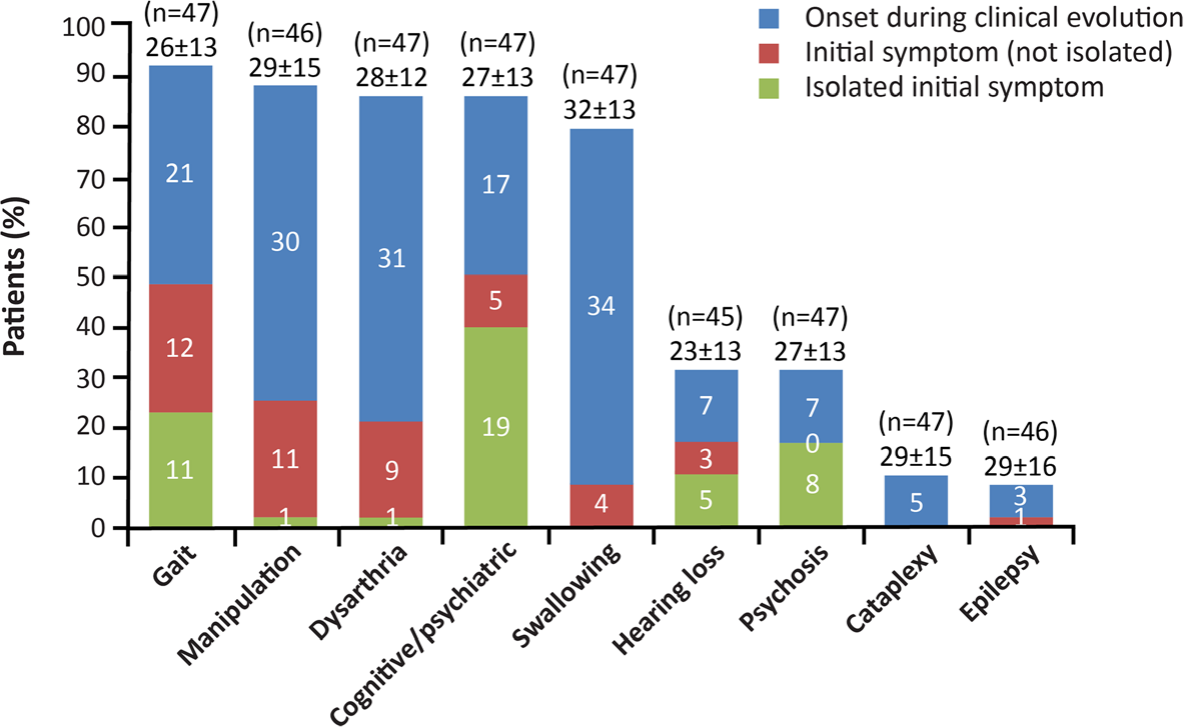
Frequencies and timings of key neurological symptoms. Color-coded bars represent symptom occurrence (% patients) as initial isolated neurological symptoms, initial neurological symptoms (not isolated), or appearance during the course of neurological deterioration. This classification did not take into account vertical supranuclear gaze palsy (VSGP), cognitive developmental symptoms, or hearing loss (except for the hearing loss item). Cognitive and psychiatric symptoms were considered as a single category as they frequently overlap, and separating them according to age at onset can be arbitrary. Psychosis is contained within the Cognitive/Psychiatric category, but is also shown as a separate item due to its particular importance among adult/adolescent patients. N numbers above each bar are total numbers of patients analysed for each symptom. Mean ± SD ages at onset of each symptom are shown above each bar

Impaired gait, cognitive/psychiatric symptoms, impaired manipulation, dysarthria, and dysphagia were the most frequent neurological symptoms, observed in 81–94% of patients overall (Fig. 2 and Additional file 2: Figure S1**)**. In particular, impaired gait and cognitive/psychiatric symptoms very often featured as initial disease manifestations, sometimes in isolation. Gait disorder was mainly due to cerebellar ataxia (*n* = 40), and less often to generalized dystonia (*n* = 15), myoclonus (*n* = 3), and lower-limb spasticity (*n* = 7, never prominent). Cognitive decline, assessed by low performance on the Mini Mental State Examination (MMSE) and/or the Frontal Assessment Battery (FAB) in 41/47 patients was associated with behavioural signs of frontal syndrome (apathy, intolerance to frustration, disinhibition) in 14/41 patients. Detailed cognitive impairments for a subset of these cognitively impaired patients were reported by Heitz et al. in 2017 [41]. Schizophrenia-like psychosis featuring delusions and hallucinations was observed in 32% of patients, and occurred as the only initial disease manifestation in over half of the cases in whom it was recorded.

Overall, 20/46 patients (43%) presented with a single isolated neurological or psychiatric manifestation without any other previous disorder. Impaired manipulation, dysarthria and dysphagia were rarely featured among the initial manifestations. VSGP was present in almost all patients (94%), but the age at onset of this manifestation was generally not measureable as it was mainly detected through clinical examination rather than patient report. Hearing loss was observed in 32% of patients, and sometimes preceded motor and/or cognitive symptoms.

### Miglustat treatment

Thirty-seven of the 47 patients in the cohort (79% overall) received miglustat, which was not available for patients who received care before 2006. The mean ± SD period between neurological onset and initiation of miglustat treatment was 11 ± 8.7 years (range 1.0–48.0 years). Among patients with available data who continued treatment throughout the observation period (*n* = 28), the mean ± SD duration of miglustat therapy was 3.4 ± 3.1 years (range, 0–9.8 years). Among those who discontinued miglustat during the observation period (*n* = 8), the mean ± SD duration of therapy was 0.9 ± 0.9 years (range 0.2–2.8 years). All but three patients who received miglustat had been diagnosed less than 13 months before starting treatment.

### NP-C disability scores

**Fig. 3:**
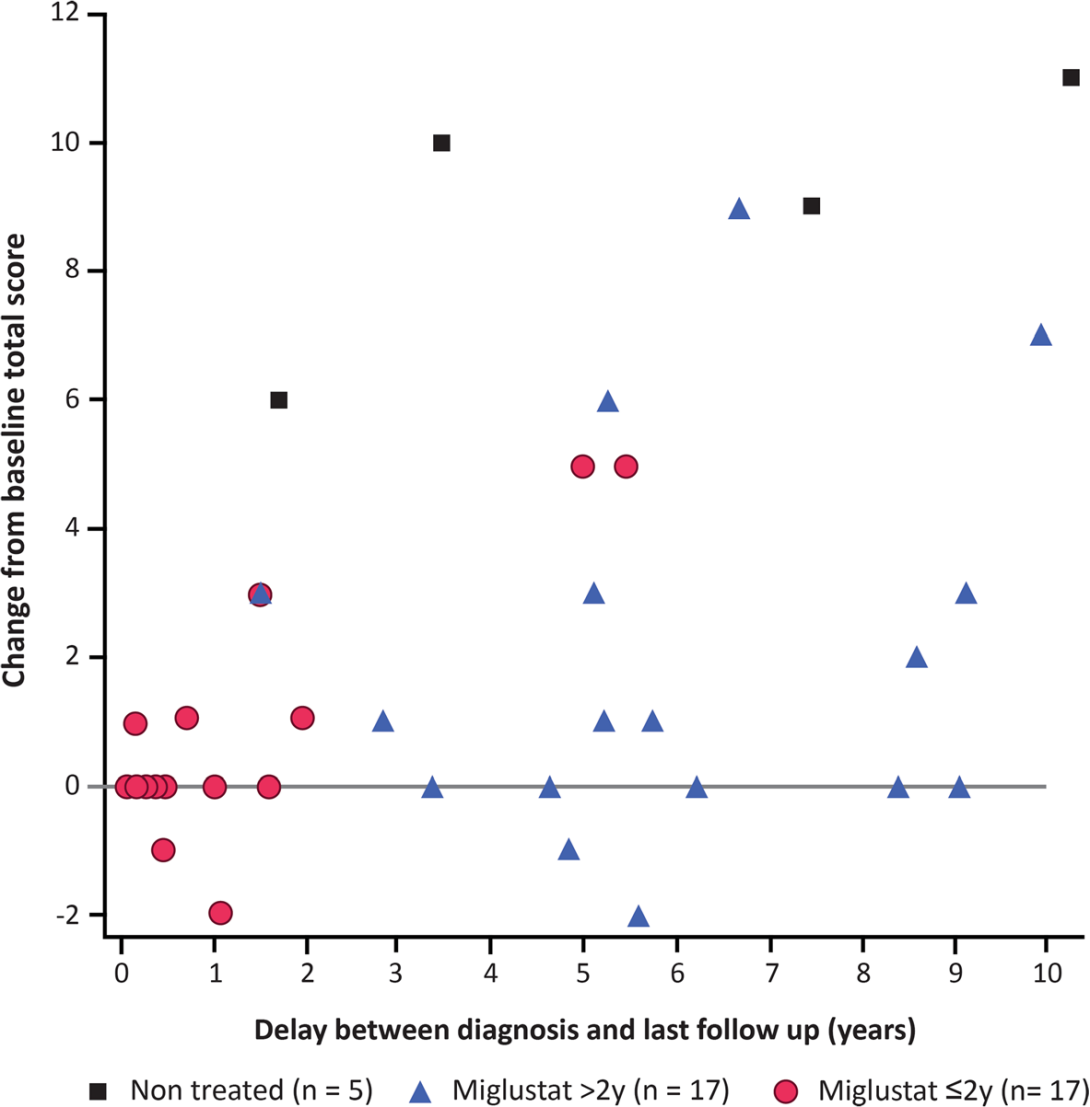
Changes in total NP-C disability score for each patient from baseline (diagnosis) to last follow up. Each point represents change in total disability score in individual patients according to delay between diagnosis and last follow up. A positive change in disability score indicates clinical worsening. Patients who discontinued miglustat after < 2 years due to neurological worsening were excluded (*n* = 4). For three patients (2, 6, and 16), change in disability score was measured between age at miglustat onset and age at last examination, as delay between diagnosis and miglustat onset exceeded 1 year. The period between diagnosis and last follow up and the duration of miglustat treatment were associated with change in disability score from baseline (*p* < 0.001 for both variables). Clinical score at diagnosis and age at neurological onset did not show any statistically significant relationship (*p* = 0.34 and 0.30, respectively)

**Fig. 4:**
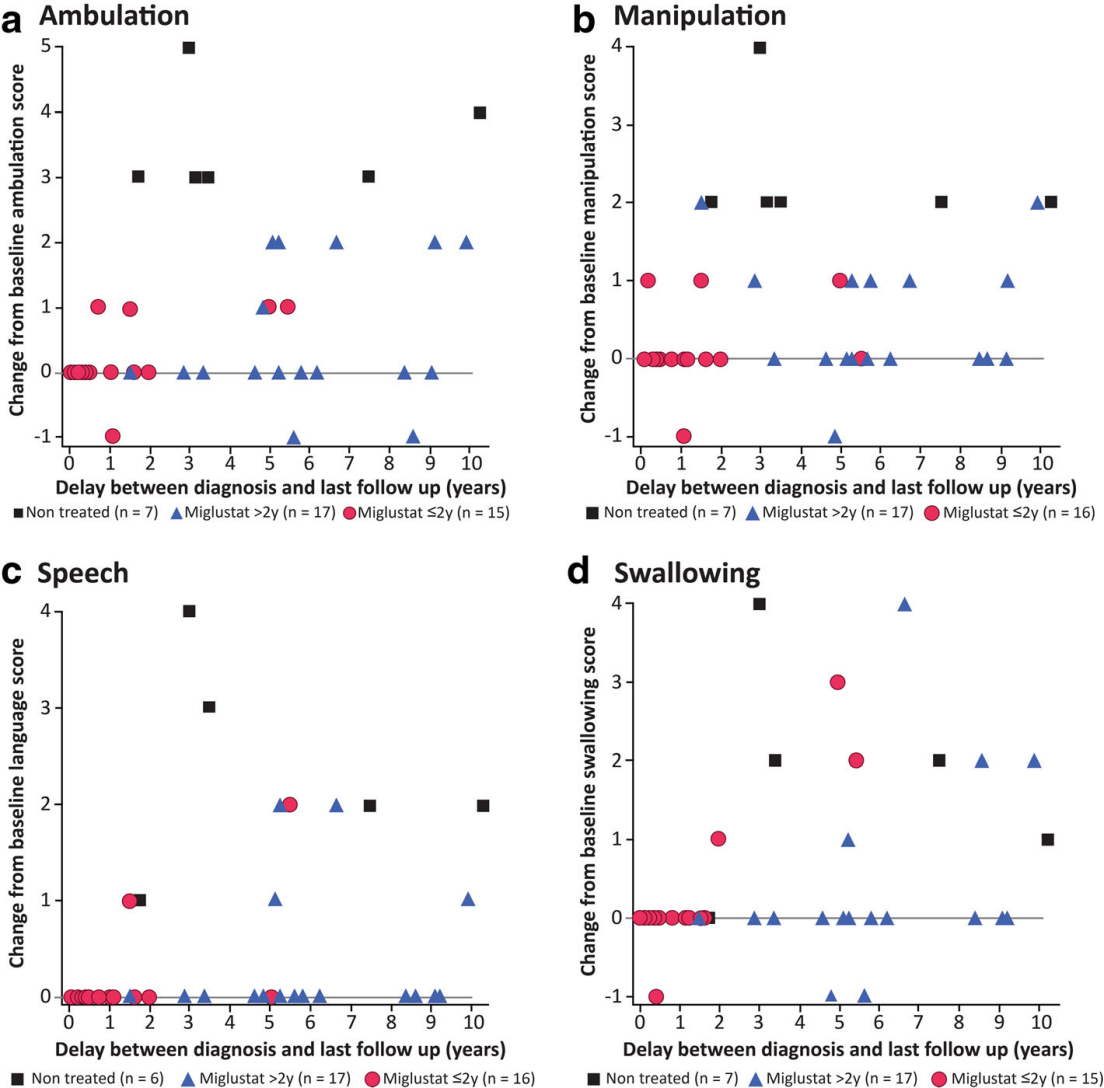
Change in individual NP-C disability subscores for each patient from baseline (diagnosis) to last follow up. Changes in gait (panel **a**; *p* < 0.001), manipulation (panel **b**; *p* = 0.016), speech (panel **c**; *p* < 0.001) and swallowing subscores (panel **d**; *p* = 0.0176) were statistically significantly associated with duration of miglustat treatment

Patients who received miglustat for > 2 years worsened less than untreated patients or those treated for < 2 years (Fig. 3). Regression analysis revealed strong correlations for change in disability score with both duration of miglustat treatment (*p* < 0.001) and delay from diagnosis to last follow up (*p* < 0.001). Age at neurological onset and disability score at diagnosis were not associated with change in disability score (*p* = 0.30 and *p* = 0.34, respectively). In identical statistical analyses of scores for each disability scale subscore, duration of miglustat treatment showed statistically significant associations with changes in subscores for gait (*p* < 0.001), manipulation (*p* = 0.005), speech (*p* < 0.001), and swallowing (*p* = 0.04) (Fig. 4). For treated patients at diagnosis (< 1 year between diagnosis and miglustat start), we identified ‘poor responders’ as those who had an increase in total score of > 2 despite more than 2 years of miglustat (*n* = 5) or who stopped miglustat before 2 years because of neurological worsening (*n* = 4), and ‘good responders’ as those who did not increase their total clinical score to > 2 after at least 2 years of miglustat (*n* = 10). The clinical characteristics of these two subgroups are summarized in Table 2. The mean composite NP-C disability score at start of miglustat treatment was lower in good responders (8.8) than in poor responders (13.0) (*p* = 0.021).

**Fig. 5:**
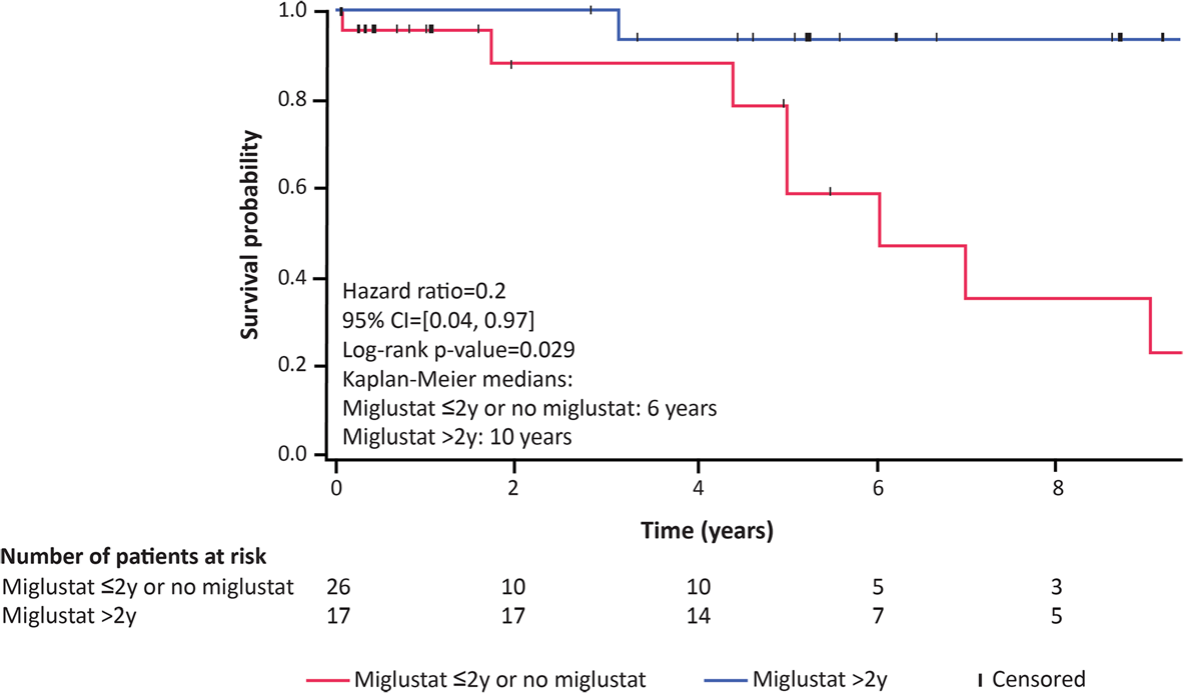
Time-to-event analysis of period from diagnosis to death in patients treated with miglustat for > 2 years (*n* = 17) versus untreated patients and those who received miglustat for < 2 years (*n* = 26). Patients who discontinued miglustat after < 2 years of treatment because of neurological worsening were excluded (*n* = 4). The Kaplan Meier curves for this analysis were truncated when approximately 10% of patients were still under observation in each group, due to low relevance of graphical representation based on limited patient numbers beyond this time point. For patient 6 who was diagnosed in early infancy, time-to-event analysis began from start of miglustat treatment. Mean clinical scores at diagnosis were not different between the two groups (9.4 in patients treated for > 2 years versus 9.1 in untreated patients and those receiving miglustat for < 2 years). A statistically significant delay to death was noted in patients treated with miglustat for > 2 years versus untreated patients and those receiving miglustat for < 2 years (*p* = 0.029; log-rank test)

**Fig. 6:**
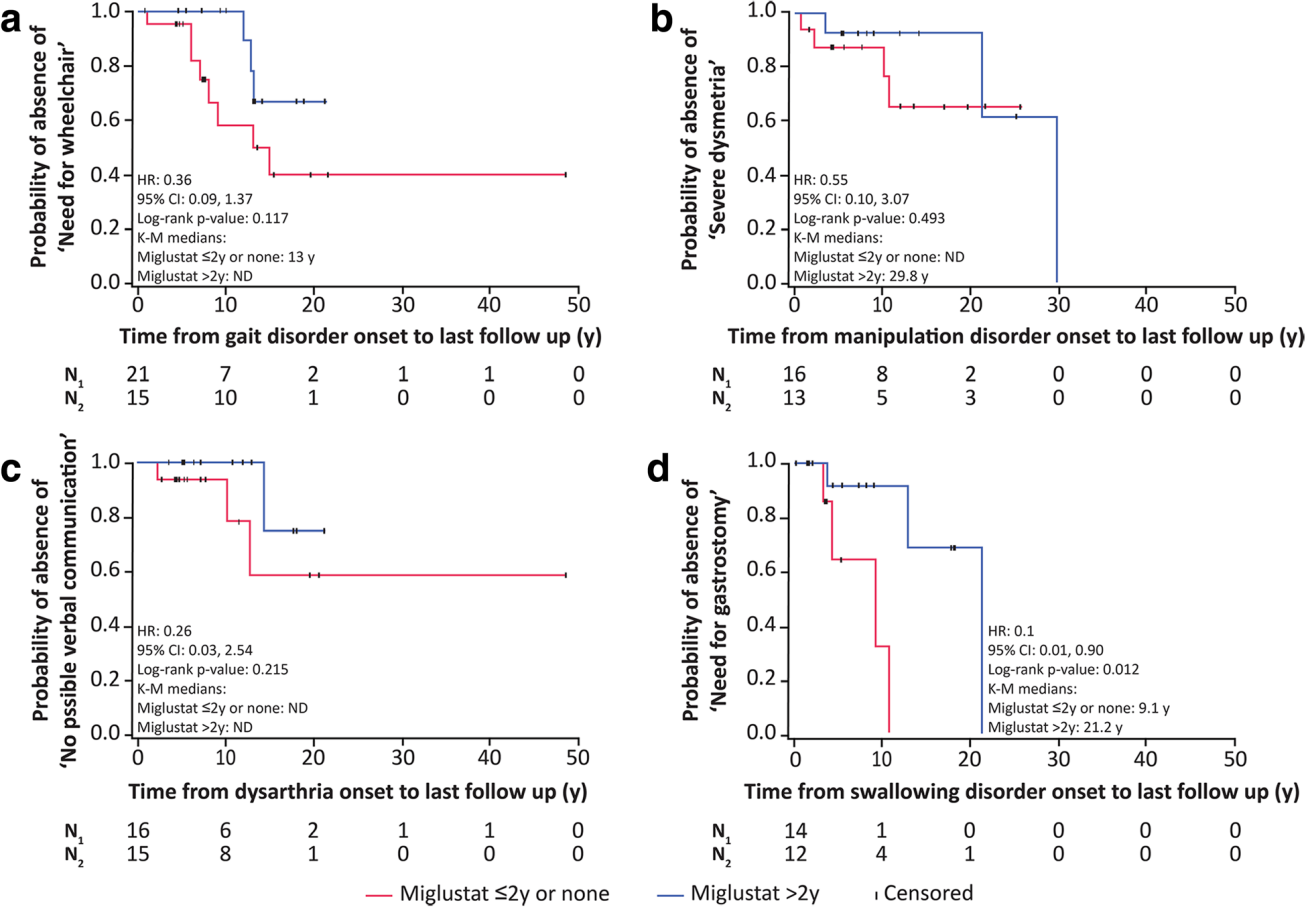
Time-to-severe-event analysis for: a) impaired gait (need for wheelchair), b) manipulation (severe dysmetria); c) speech (non-verbal communication); and d) swallowing (need for gastrostomy) subscores in patients treated with miglustat for > 2 years versus untreated patients and those receiving miglustat for < 2 years. N_1_, number of patients untreated or receiving miglustat for ≤2 years; N_2_, number of patients treated with miglustat for > 2 years; y, years

Kaplan-Meier survival analysis of time to death comparing 1) patients who received > 2 years of miglustat therapy with 2) untreated patients and those who received < 2 years of treatment indicated a statistically significant increase in ‘Time to death’ with > 2 years of therapy (*p* = 0.029) (Fig. 5). Similarly, time-to-event analyses comparing these two treatment groups for individual domain items indicated increased ‘Time to reach the most severe disability category’ per domain, although statistical significance was only observed for ‘Need for gastrostomy’ (*p* = 0.012) (Fig. 6).

### Safety and tolerability findings

Among 37 miglustat-treated patients, 36 adverse events in 29 patients were attributed to miglustat due to their occurrence after miglustat initiation: weight loss (*n* = 17), diarrhoea (*n* = 12), upper-limb tremor (*n* = 3), depression (*n* = 2), elevated hepatic transaminases (n = 1), and onset of a first psychotic episode (*n* = 1).

In total, eight patients (22%) discontinued miglustat during the observation period. Three discontinued due to adverse events. One stopped treatment due to severe weight loss. The patient with elevated hepatic transaminase activity showed a seven-fold increase in aspartate amino transferase (AST) and an eleven-fold increase in alanine amino transferase (ALT) activities that were considered by the treating physician as possibly related to miglustat because: 1) they occurred a few weeks after miglustat initiation; 2) no other aetiology was found, and; 3) activities normalized after miglustat discontinuation. The first psychotic episode was also considered by the treating psychiatrist as likely related to miglustat because: 1) it occurred a few weeks after miglustat initiation; 2) psychotic symptoms stopped after miglustat discontinuation; 3) psychotic symptoms re-occurred after re-initiation of miglustat; and 4) 6 years after definitive discontinuation of miglustat, the patient had never relapsed. Perceived lack of miglustat efficacy, with ongoing neurological worsening, was cited as the reason for discontinuation in five patients, among whom four showed continued worsening after discontinuation and one was lost to follow up.

## Discussion

No adult-onset NP-C cases were diagnosed in France until 1990; the 6% of adult cases reported in an early survey of 125 patients studied in the French reference laboratory originated from other European countries, particularly Germany [14]. In the present study, most patients with a late-onset neurological form were diagnosed after 2008. It is noticeable that during the period 2009–2015, NP-C diagnoses in France were as frequent among adults as in paediatric-onset cases. In contrast, adult cases represented only one fifth of all cases diagnosed during the period 2000–2008 (personal data from PL and MTV). This suggests a very significant improvement in awareness of NP-C among neuropsychiatrists after miglustat therapy became available. Use of diagnostic plasma biomarkers cannot explain this recent increase in diagnosed adult cases, as they were not routinely implemented in France until 2015.

Profiling of neurological manifestations over the course of the disease identified four main initial clinical phenotypes: 1) gait disorder with cerebellar ataxia and/or dystonia (in 49% of patients); 2) cognitive and behavioural disorder due to frontal syndrome (in 34%); 3) psychosis mimicking schizophrenia (in 17%); and 4) hearing loss (in 18%). The three first early phenotypes have previously been described [5, 20, 21, 42, 43] but to our knowledge hearing loss – although a known feature of the disease [4, 44, 45] – has never been reported as a presenting sign, possibly because the timing of onset was not investigated. However, based on Brainstem Auditory Evoked Potentials (BAEP) studies it has been suggested that the auditory pathway is consistently affected in the adult-onset form of NP-C [46]. Of note, auditory testing in *Npc1*^nih^ mutant mice revealed an early progressive high frequency hearing loss that occurred before overt neurological symptoms [47].

While these phenotypes may overlap, in this study almost half of patients presented with a single isolated neurological or psychiatric manifestation without any other previous disorder. This leads to challenging diagnostic work up. However, the early diagnosis of NP-C is crucial for effective disease management. The use of miglustat in this cohort of adolescent/adult-onset patients slowed the progression of neurological manifestations, stabilizing some patients for several years, especially those who benefited from miglustat whereas their disability was still moderate.

Only a minority of patients in this cohort had paediatric signs of the disease that can be divided into three types: 1) clinical hepatomegaly and/or splenomegaly (observed in 7/40 of our patients); 2) cognitive developmental symptoms (in 11/45 patients); and 3) early onset of neurodegenerative manifestations at < 15 years of age (i.e., juvenile onset).

It is recognized that clinical hepatomegaly and splenomegaly do not correlate with the severity of neurological symptoms in NP-C [2, 5]. In support of this, two patients from the overall French cohort, diagnosed in early childhood based on systemic symptoms (hepatomegaly and/or splenomegaly and/or neonatal cholestasis), did not have neurological symptoms at last follow up despite their now being 24- and 16-years old. A third patient (patient 6, Fig. 1, Additional file 1: Table S1) was diagnosed at 3 months of age and did not show neurological signs until the age of 12 years. Previous studies have also reported that hepatomegaly and/or splenomegaly are often present in adolescent/adult-onset NP-C but are usually so mild that they can only be detected during echography [6, 10, 21]. This was also supported by our data.

To our knowledge there are only anecdotal data from case reports and case series that have previously examined developmental cognitive deficits alongside subtle motor signs (not specifically reported here) as possible prodromal signs of NP-C in the longitudinal course of adolescent/adult-onset NP-C [19, 48, 49]. In contrast, developmental delay and psychomotor regression are widely reported and acknowledged as early manifestations in paediatric-onset NP-C [2, 6, 11].

Early onset of neurodegenerative disease (at < 15 years of age, i.e. classical juvenile onset) sometimes overlaps with prodromal signs, which complicates assessment of the age at neurological onset. The 10 ‘late juvenile/early adolescent’-onset patients in the current cohort, which included only patients who reached adulthood, have a phenotype closer to the adult-onset form of NP-C than the classical juvenile-onset form, especially in terms of prognosis. Such patients might best be described as having the adolescent/adult-onset form in a revised version of the usual classification. This indicates that for patients who show overlap in terms of the usual age at onset of the ‘classical’ neurological forms, the *type* of initial neurological signs as well as the *rate* of further neurological deterioration should be considered. This also applies to the other neurological forms. It should be kept in mind that when the classification was first described [14, 50], the age of neurological onset was defined as the time when the first symptom typical of NP-C was noted, often retrospectively based on medical records. Prodromal signs or signs not considered then as frequent early signs of NP-C were not factored in.

VSGP was present in almost all patients in this cohort. However, it was usually first observed several years after neurological disease onset, which raises as yet unanswered questions regarding the precocity of this neurological manifestation. As the filipin test is invasive, time consuming, and can be costly, clinicians tended to request it only in patients with a high suspicion of NP-C – very often only those who display VSGP. Historically, this diagnostic bias probably led to an overestimation of VSGP frequency in studied cohorts, including ours. From now on, the widespread use of newly available non-invasive diagnostic biomarkers (e.g. plasma oxysterols or lysosphingolipids [3, 30]) will likely identify NP-C patients with no VSGP at diagnosis, as we have begun to observe.

In recent years, a larger number of recurrent *NPC1* and *NPC2* mutations have been described worldwide, and some genotype/phenotype associations have emerged. From the present and other studies, patients with V950 M, R978C, G992R, D874V mutations in one *NPC1* allele have so far shown an adolescent/adult neurological form of NP-C, even in association with a very severe allele (e.g., in patients 3, 6, 7, 8, 11, 16, 29, 31, 32, 33, 36 and 46) [6, 21, 51, 52].

The retrospective collection of clinical data is a limitation in this study. However, the gross nature of clinical score assessments based on easily identified steps in our patients helped to limit the possibility of retrospective scoring errors. Another study limitation is that many patients who were not treated with miglustat were diagnosed in the 1990s, and may therefore not have benefited from the same quality of care. It is also worth noting that the disability scale data reported here only describe motor symptoms, while cognitive and psychiatric symptoms, which are more difficult to analyse retrospectively, also have a strong effect on patient quality of life. Additionally, the ever-changing landscape of symptomatic treatments for psychotic symptoms in psychiatric practice can create further complications for assessing the efficacy of miglustat.

Previous studies have evaluated the long-term effects of miglustat in NP-C and have shown that affected adults benefited from miglustat therapy more than children, displaying prolonged neurological stabilization in many cases [28, 35, 36]. Cognition, which was not specifically analysed in the current study, was reported in a subset of miglustat-treated patients from the current cohort who globally remained stable, even after several years of follow up in some cases [41]. However, cognitive worsening during miglustat therapy has also been reported in other long-term assessments [36, 53].

Our present investigation, which features more adult patients and longer follow up than previous reports, indicates that miglustat has a positive impact on long-term disease course, slowing-down neurological deterioration, delaying the occurrence of disease severity milestones and, ultimately, prolonging survival in adults. A positive effect of miglustat on patient survival, possibly related to improvements in swallowing, has been reported previously [54]. However, beneficial effects were not observed in all patients in our analysis. Severe motor disability (including gait, manipulation, dysarthria, and swallowing disorder) at miglustat initiation appeared to have a negative effect on response to treatment, with some patients showing continued neurological deterioration despite therapy (all patients with a disability scale score > 12 were poor responders to miglustat). A better response to miglustat in patients with less severe disease at treatment initiation, as seen here, has previously been reported [29]. Since neurological disease severity at treatment initiation is not an absolute predicting factor for therapeutic response, miglustat therapy should probably be attempted in all patients with adult-onset NP-C. However, miglustat should be stopped early if neurological worsening is observed, especially in severely disabled patients (disability scale score > 12).

## Conclusions

The detection of NP-C among adults in France greatly improved over the last decade, particularly since 2009 due primarily to increased awareness. A small percentage of patients who had presented in the juvenile period with non-specific, very mild neurological symptoms have shown a slow further course of disease different from that of the classical juvenile neurological form, advocating a wider concept of the adolescent/adult-onset form. Our analyses of long term follow up of miglustat therapy indicated that this pharmacological agent has had a positive impact on disease course in many patients, globally slowing down neurological deterioration and prolonging survival, although not in patients who initiated treatment at a late stage of neurological disability.

## Additional files

Additional file 1: **Table S1.** Individual patient data summary. (DOCX 33 kb)

Additional file 2: **Figure S1.** Time-to-event analysis for period between neurological onset and appearance of neurological manifestations of interest. VSGP and hearing loss were not taken into account in determining neurological onset. Cognitive and psychiatric symptoms were considered as a single category because they frequently overlap and their separation according to respective ages at onset may be arbitrary. Psychosis, which is part of the ‘Cognitive/Psychiatric’ category, was also analysed separately. (TIF 1229 kb)

## Abbreviations

ALT: Alanine amino transferase
AST: Aspartate amino transferase
CRML: Reference Center for Lysosomal Diseases
FAB: Frontal Assessment Battery
HSMG: hepatosplenomegaly
ID: Intellectual disability
LD: Learning disabilities
MMSE: Mini Mental State Examination
NP-C: Niemann-Pick disease type C
*NPC1/NPC2*: Genes encoding the NPC1/NPC2 proteins
SI: Suspicion index
VSGP: Vertical supranuclear gaze palsy
