# Supplementary material for: Adult Niemann-Pick disease type C in France: clinical phenotypes and long-term miglustat treatment effect
Source: Orphanet J Rare Dis. 2018 Oct 1;13:175. doi: 10.1186/s13023-018-0913-4 (PMC6167825; doi:10.1186/s13023-018-0913-4)
Supplement: Supplementary file 1 — Table S1. Individual patient data summary. (DOCX 33 kb) [file 13023_2018_913_MOESM1_ESM.docx]

**Supplementary Table S1.** Individual patient data summary

| **Patient number (previous reference)** | **Age at neurol. onset (years)^a^** | **Age at diagnosis (years)** | **Age at death (years)** | **Age at last FU (years)** | **Age at miglustat start (years)** | **Miglustat treatment duration (months)** | **Miglustat discontinued  (Y = yes)** | **Total score^c^ at diagnosis** | **Total score^c^ at miglustat start** | **Total score^c^ at last FU** | **Filipin test finding** | **Gene mutations**  **(all *NPC1* except #42)** |
| --- | --- | --- | --- | --- | --- | --- | --- | --- | --- | --- | --- | --- |
| 1 [1] | 8 | 15 | NR | 21 | 15 | 70 | – | 12 | 12 | 12 | CLA | R589C / R589C |
| 2 [2] | 10 | 18 | 31 | 31 | 21 | 118 | – | 9 | 10 | 17 | VAR | G992A / G993fs |
| 3 [1, 2] | 12 | 22 | NR | 31 | 22 | 101 | – | 10 | 10 | 13 | VAR | I1061T / V950M |
| 4 | 12 | 37 | NR | 38 | 37 | 4 | Y^b^ | 19 | 19 | 19 | NA | Q119fs / (Spli E1)^d^ |
| 5 [3] | 18 | 26 | NR | 26 | 26 | 0 | – | 11 | 11 | 11 | INT | Q119fs / (Spli E1)^d^ |
| 6 [1] | 12 | 0 | NR | 24 | 15 | 102 | – | 0 | 7 | 9 | VAR | G992R / (partial retention intron 1)^e^ |
| 7 [1, 2] | 13 | 17 | 32 | 32 | 27 | 12 | Y^b^ | 5 | 14 | 17 | VAR | I1061T / V950M |
| 8 [1] | 13 | 22 | NR | 27 | 22 | 58 | – | 10 | 10 | 11 | INT | I1061T / V950M |
| 9 [2] | 14 | 25 | 29 | 29 | NR | NR | – | 7 | NR | 13 | CLA | Y871C / F763L |
| 10 [1] | 14 | 21 | NR | 27 | 22 | 34 | Y^b^ | 16 | 16 | 22 | CLA | I1061T / G548A |
| 11 [1] | 14 | 22 | NR | 28 | 23 | 60 | – | 8 | ND | 17 | INT | R978C / M631R |
| 12 [1, 4] | 15 | 23 | NR | 28 | 23 | 59 | – | 9 | 10 | 10 | INT | Q252fs / C800R |
| 13 | 15 | 62 | 64 | 64 | 63 | 10 | – | 10 | 10 | 13 | VAR | S344fs / D531N |
| 14 | 56 | 60 | NR | 60 | 60 | 5 | – | 8 | 8 | 7 | INT | S344fs / D531N |
| 15 [1, 2] | 16 | 20 | NR | 29 | 20 | 104 | – | 10 | 10 | 10 | CLA | I1061T / G538R |
| 16 [1, 2] | 16 | 28 | NR | 46 | 37 | 103 | – | 10 | 15 | 15 | VAR | G992R / N452fs |
| 17 [2] | 16 | 24 | NR | 36 | NR | NR | – | 11 | NR | 20 | CLA | K592fs / R615L |
| 18 [1] | 16 | 36 | NR | 42 | 37 | 13 | Y^b^ | 9 | 9 | 20 | VAR | P1007A / P1007A |
| 19 [1] | 32 | 33 | NR | 38 | 33 | 5 | Y | 2 | 2 | 7 | VAR | P1007A / P1007A |
| 20 | 16 | 32 | NR | 35 | 32 | 30 | – | 5 | 5 | 6 | NA | V1158M / V1158M |
| 21 | 16 | 23 | NR | 24 | 23 | 10 | – | 7 | 7 | 5 | INT | V1158M / V1158M |
| 22 | 16 | 24 | NR | 26 | 25 | 10 | – | 1 | 1 | 1 | VAR | V664M / S1200G |
| 23 [2] | 17 | 29 | 34 | 34 | NR | NR | – | NA | NR | NA | CLA | R1059Q / L724P |
| 24 [2] | 18 | 33 | 40 | 40 | NR | NR | – | 12 | NR | 22 | CLA | S344fs / R372W |
| 25 [4] | 18 | 29 | 33 | 33 | 30 | 36 | – | 14 | 14 | 17 | INT | I1061T / ?(V562V)^f^ |
| 26 [4] | 18 | 35 | NR | 40 | 36 | 52 | – | 5 | 5 | 5 | INT | I1061T / ?(V562V)^f^ |
| 27 [1] | 20 | 25 | NR | 29 | 25 | 51 | – | 8 | 8 | 7 | INT | S954L / N1186S |
| 28 | 20 | 28 | NR | 29 | 28 | 10 | – | 17 | 17 | 16 | NA | Y475C / (splice exon 2)^g^ |
| 29 | 23 | 27 | NR | 32 | 28 | 51 | – | 12 | 12 | 15 | INT | R978C / del exon 4^h^ |
| 30 | 23 | 32 | NR | 32 | 32 | 6 | – | 11 | 11 | 12 | VAR | P1007A / P474L |
| 31 [1] | 25 | 41 | NR | 46 | 41 | 62 | – | 10 | 10 | 8 | INT | G992R / Y1081X |
| 32 [5, 6] | 25 | 27 | NR | 28 | 27 | 11 | – | NA | NA | 3 | NA | R978C / del exons 1-6^h^ |
| 33 [5, 6] | NR | 22 | NR | 22 | 22 | 2 | – | 2 | 2 | 2 | NA | R978C / del exons 1-6^h^ |
| 34 | 25 | 28 | NR | 30 | 28 | 21 | – | 11 | 11 | 12 | INT | R1186H / V757M |
| 35 [1] | 27 | 36 | NR | 41 | 36 | 6 | Y | 7 | 7 | 12 | CLA | C227S / I841K |
| 36 [2] | 27 | 40 | 49 | 49 | NR | NR | – | NA | NR | 7 | VAR | V950M / V950M |
| 37 [2] | 28 | 32 | 37 | 37 | NR | NR | – | NA | NR | NA | CLA | I1061T / A605V |
| 38 [2] | 30 | 41 | 52 | 52 | NR | NR | – | 9 | NR | 20 | CLA | F283fs / D1097N |
| 39 | 30 | 49 | NR | 50 | 49 | 12 | – | 10 | 10 | 8 | VAR | D242N / (splice exon 19)^i^ |
| 40 [2] | 34 | 34 | 39 | 39 | NR | NR | – | NA | NR | NA | CLA | Y825C / S940L |
| 41 | 40 | 56 | NR | 57 | 56 | 8 | Y | 12 | 12 | 13 | INT | G1012C / (splice exon 3)^j^ |
| 42 | 43 | 46 | NR | 46 | 46 | 0 | – | 5 | 5 | 5 | CLA | ***NPC2*** P120S / P120S |
| 43 | 44 | 59 | NR | 62 | 60 | 26 | – | 11 | 11 | 11 | VAR | P1007A / R615C |
| 44 | 44 | 52 | NR | 54 | 53 | NA | NA | NA | NA | NA | INT | G61K / S636F |
| 45 | 50 | 54 | NR | 54 | 54 | 2 | Y^b^ | 10 | 10 | 10 | INT | Y146N / G910S |
| 46 [4] | 50 | 59 | 59 | 59 | NR | NR | – | NA | NR | NA | CLA | D874V / F66fs |
| 47[3] | 50 | 65 | NR | 65 | NR | NR | – | 10 | NR | 10 | NA | S386G / M631R |

*^a^Not including VSGP and hearing loss.*

*^b^Patient stopped miglustat because of perceived lack of efficiency.*

*^c^Based on disability scale [7]. Patients numbers (4;5), (13;14), (18;19), (20;21), (25;26) and (32;33) are siblings.*

*Abbreviations: CLA, classic; FU, follow up; INT, intermediate; NR, not relevant; NA, not available; VAR, variant.*

Additional information regarding indicated *NPC1* mutations: patients 4 and 5 ^d^[c.57+4A>G]; 6 ^e^[c.58-3290G>A]; 25 and 26 ^f^[c.1686G>A]; 28 ^g^[c.181-6T>A]; 29, 32, and 33 ^h^by multiplex ligation-dependent probe amplification (MLPA); 39 ^i^[c.2796-12_2796-9del]; 41 ^j^[c.181-2A>G]. Reference sequence NM_000271.4.

**References**

1. Sedel F, Chabrol B, Audoin B, Kaphan E, Tranchant C, Burzykowski T, Tourbah A, Vanier MT, Galanaud D. Normalisation of brain spectroscopy findings in Niemann-Pick disease type C patients treated with miglustat. J Neurol. 2016;263**:**927-36.

2. Sevin M, Lesca G, Baumann N, Millat G, Lyon-Caen O, Vanier MT, Sedel F. The adult form of Niemann-Pick disease type C. Brain. 2007;130**:**120-33.

3. Marelli C, Guissart C, Hubsch C, Renaud M, Villemin JP, Larrieu L, Charles P, Ayrignac X, Sacconi S, Collignon P, et al. Mini-Exome Coupled to Read-Depth Based Copy Number Variation Analysis in Patients with Inherited Ataxias. Hum Mutat. 2016;37**:**1340-53.

4. Anheim M, Lagha-Boukbiza O, Fleury-Lesaunier MC, Valenti-Hirsch MP, Hirsch E, Gervais-Bernard H, Broussolle E, Thobois S, Vanier MT, Latour P, Tranchant C. Heterogeneity and frequency of movement disorders in juvenile and adult-onset Niemann-Pick C disease. J Neurol. 2014;261**:**174-9.

5. Abela L, Plecko B, Palla A, Burda P, Nuoffer JM, Ballhausen D, Rohrbach M. Early co-occurrence of a neurologic-psychiatric disease pattern in Niemann-Pick type C disease: a retrospective Swiss cohort study. Orphanet J Rare Dis. 2014;9**:**176.

6. Stampfer M, Theiss S, Amraoui Y, Jiang X, Keller S, Ory DS, Mengel E, Fischer C, Runz H. Niemann-Pick disease type C clinical database: cognitive and coordination deficits are early disease indicators. Orphanet J Rare Dis. 2013;8**:**35.

7. Pineda M, Perez-Poyato MS, O'Callaghan M, Vilaseca MA, Pocovi M, Domingo R, Ruiz Portal L, Verdu Perez A, Temudo T, Gaspar A, et al. Clinical experience with miglustat therapy in pediatric patients with Niemann-Pick disease type C: A case series. Mol Genet Metab. 2010;99**:**358-66.
