# Supplementary figures and images for: Adult Niemann-Pick disease type C in France: clinical phenotypes and long-term miglustat treatment effect
Source: Orphanet J Rare Dis. 2018 Oct 1;13:175. doi: 10.1186/s13023-018-0913-4 (PMC6167825; doi:10.1186/s13023-018-0913-4)

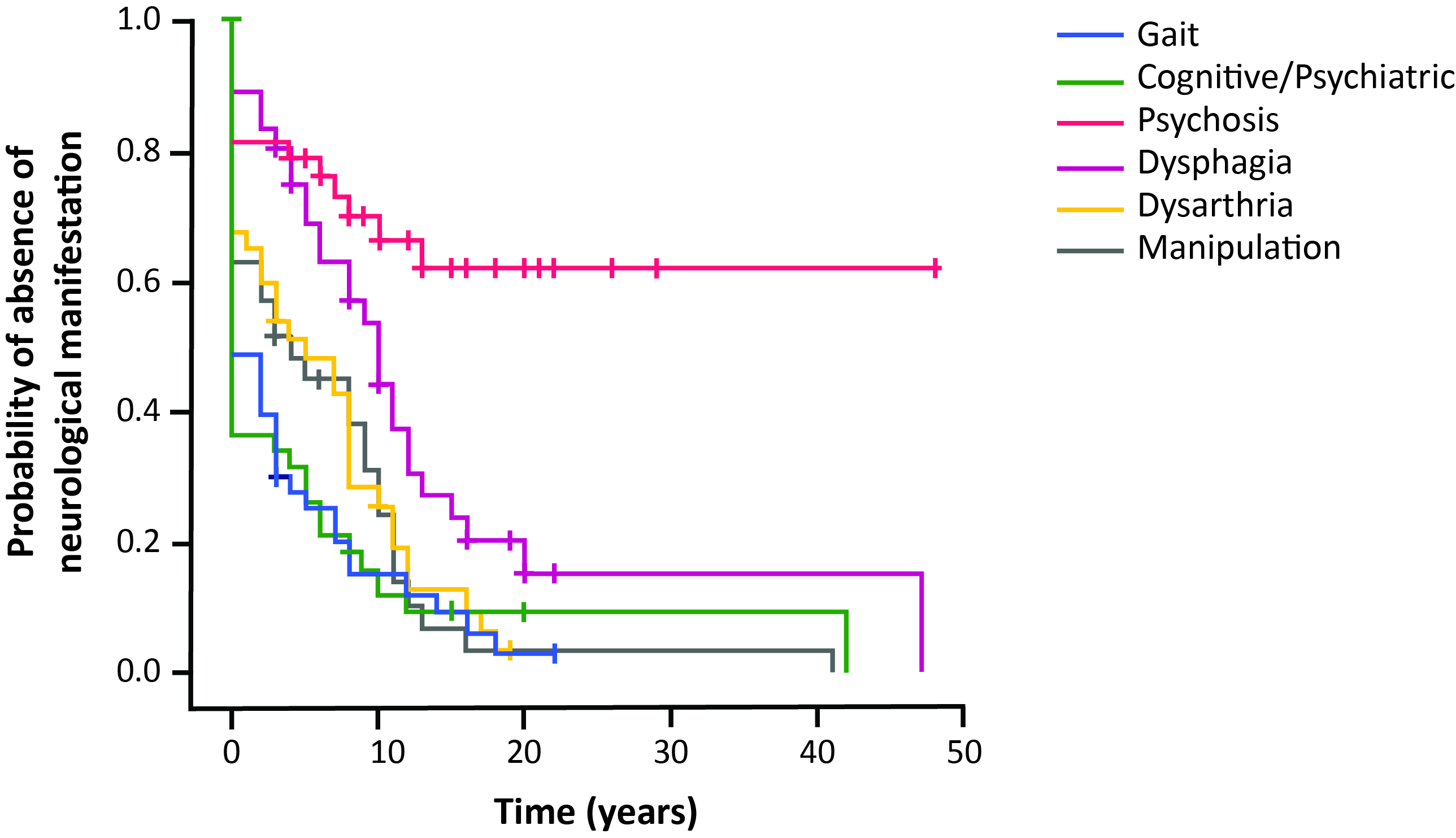

Supplement: Supplementary file 2 — Figure S1. Time-to-event analysis for period between neurological onset and appearance of neurological manifestations of interest. VSGP and hearing loss were not taken into account in determining neurological onset. Cognitive and psychiatric symptoms were considered as a single category because they frequently overlap and their separation according to respective ages at onset may be arbitrary. Psychosis, which is part of the ‘Cognitive/Psychiatric’ category, was also analysed separately. (TIF 1229 kb) [file 13023_2018_913_MOESM2_ESM.tif]
